# Adjuvant is necessary for a robust immune response to a single dose of H1N1 pandemic flu vaccine in mice

**DOI:** 10.1371/currents.RRN1025

**Published:** 2009-09-01

**Authors:** Philip R. Dormitzer, Rino Rappuoli, Daniele Casini, Derek O'Hagan, Celene Runham, Emanuele Montomoli, Barbara Baudner, John J. Donnelly III, Giulia Lapini, Andreas Wack

**Affiliations:** ^*^Novartis Vaccines and Diagnostics, Cambridge, MA, USA; ^†^Novartis Vaccines & Diagnostics; ^§^Novartis Vaccines; ^#^University of Siena; ^**^Novartis and ^§§^Division of Immunoregulation, National Institute for Medical Research, The Ridgeway, London NW7 1AA

## Abstract

Pandemic H1N1 influenza vaccine antigens are currently being manufactured. The MF59 adjuvant has an established safety profile in humans and a proven ability to increase responses to some influenza vaccines in humans. To inform initial decisions on the use of these vaccine components to protect human populations, we have immunized mice with MF59-adjuvanted or non-adjuvanted pandemic influenza vaccine. Immunizing unprimed mice with a single dose of MF59-adjuvanted vaccine elicits functional antibody titers equivalent to those associated with protection of humans from seasonal influenza. Without adjuvant, two doses are required for a robust antibody response. Unadjuvanted vaccines with 0.5 and 1 microgram of antigen elicit equivalent titers. These data support including MF59 in pandemic flu vaccines to rapidly protect young adults and children, who may have little or no previous exposure to influenza infection or immunization.

## Introduction 

A type A/H1N1 influenza strain, first isolated in April, 2009 from patients in Mexico and the United States with respiratory illness, has spread globally.  On June 12 the WHO declared a  pandemic.  The pandemic strain is more antigenically similar to swine influenza strains than to seasonal H1N1 strains previously circulating in humans [1].  Some older adults (33% of those older than 60 years) but few young adults and essentially no children have pre-existing serum antibody against the pandemic strain, and seasonal immunization of young people does not elicit antibodies against this strain [2].

Pandemic vaccine antigens are now being manufactured, and adjuvants are available for use, including MF59, an oil-in-water emulsion with an excellent safety profile during 12 years of commercial use in seasonal influenza vaccines in Europe [3].  Public health agencies must decide how these vaccine components will be administered (use of adjuvant, dose, number and timing of doses, priority groups for immunization) to protect human populations most effectively.  Ongoing viral spread and the anticipated increase in pandemic influenza during the upcoming northern hemisphere flu season make these decisions urgent.  People with different prior exposures to influenza antigens respond differently to immunization.  The human population includes those previously infected by seasonal influenza viruses, those previously immunized with adjuvanted and/or non-adjuvanted seasonal influenza vaccines, and those (usually children) with no previous experience of influenza vaccines or infections.

## Results  

To provide data to guide decision making before clinical trial data are available, we immunized mice with MF59-adjuvanted or non-adjuvanted pandemic H1N1 influenza subunit antigen.  The antigen was produced using the egg-based manufacturing process for Focetria®, an MF59-adjuvanted pandemic vaccine. 

A single immunization of mice with 0.5 micrograms of antigen plus MF59 elicited an average functional antibody (hemagglutination inhibition – HI) titer of 1:63 in serum obtained two weeks  after immunization (Fig. 1).  A HI titer of 1:40 or more is associated with protection of humans from seasonal influenza [4].  A second immunization with adjuvanted vaccine two weeks later increased the average HI titer to 1:1280 in serum obtained one week after the boost.  A single immunization with antigen without adjuvant did not elicit significant HI titers.  A second immunization without adjuvant two weeks later elicited a HI titer of 1:160.  There was no significant difference in the titers elicited by immunization with 0.5 or 1.0 micrograms of antigen without adjuvant.

**Figure:**
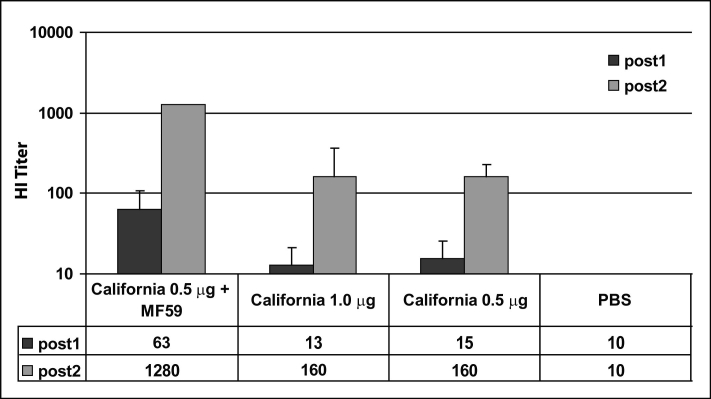
**Figure 1.  HI titers elicited by immunizing mice with pandemic H1N1 influenza vaccine antigen and MF59 adjuvant. ** Pandemic H1N1 hemagglutinin from an A/California/07/2009 H1N1-like strain (X-179A) in Focetria^®^ vaccine monobulk was quantified by single radial immunodiffusion.  Six to seven week-old Balb/c mice were immunized intramuscularly (IM) on day 0 with PBS,  with 0.5 or 1.0 microgram (HA content) of antigen alone, or with 0.5 micrograms of antigen adjuvanted with 50 microliters of MF59.  Dose volume was 100 microliters.  Sera were obtained on day 13.  Mice were boosted with a second dose, equivalent to the first, on day 14.  Sera were again collected on day 21.  Sera were assayed by HI using inactivated whole virus (X-179A) for antigen and turkey red blood cells.

## Discussion

These data obtained in mice are consistent with results of human immunization with vaccines against other potential pandemic influenza strains.  Without adjuvant, vaccines against H5 avian influenza strains elicit low antibody titers; MF59 greatly increases the rapidity, titer, and breadth of the elicited antibodies [5] [6].  During a much smaller human outbreak of swine origin influenza in 1976, adjuvanted vaccines were not available.  A single dose of the 1976 vaccines elicited low antibody titers in young people, but significantly higher titers in older individuals, probably because older subjects had experienced more priming influenza infections or immunizations [7].

The mouse immunization data support including MF59 adjuvant in H1N1 pandemic immunization campaigns, particularly for children and young adults with little or no previous exposure to influenza infection or immunization.  These individuals are particularly vulnerable to morbidity and mortality in the current pandemic [8].  With MF59-adjuvanted pandemic antigen, a single dose given to an immunologically naïve mouse produces an antibody response that is associated with protection from seasonal influenza in humans; without adjuvant, two doses are required.  In this study, no dose response was observed between 0.5 and 1 microgram of non-adjuvanted antigen.  This finding in mice increases the likelihood that dose-sparing regimens that can increase the number of available doses may prove effective in human clinical trials.  The impact of previous influenza exposures on the response to H1N1 pandemic immunization is unknown.  These mouse immunization data can inform decision-making, particularly while we await the results of pediatric immunization trials, which will lag behind trials of pandemic H1N1 influenza vaccines in adults. ****

## Acknowledgements

We thank Theodore Tsai, Giuseppe del Giudice, Christian W. Mandl, and Gene A. Palmer for helpful discussions.

## Funding Information

This study was funded by Novartis Vaccines and Diagnostics.   

## Competing Interests

BCB, CFR, JD, DC, RR, PRD, and DTO are employees and shareholders in Novartis Vaccines and Diagnostics, which manufactures influenza vaccines and adjuvants, including Focetria^®^ and MF59.
